# Medication-Related Factors and Hospital Readmission in Older Adults with Chronic Kidney Disease

**DOI:** 10.3390/jcm8030395

**Published:** 2019-03-21

**Authors:** Wubshet H. Tesfaye, Gregory M. Peterson, Ronald L. Castelino, Charlotte McKercher, Matthew Jose, Syed Tabish R. Zaidi, Barbara C. Wimmer

**Affiliations:** 1 Pharmacy, School of Medicine, College of Health and Medicine, University of Tasmania, Sandy Bay, TAS 7005, Australia; g.peterson@utas.edu.au (G.M.P.); Barbara.Wimmer@utas.edu.au (B.C.W.); 2 Sydney Nursing School, The University of Sydney, Sydney, NSW 2006, Australia; ronald.castelino@sydney.edu.au; 3 Menzies Institute for Medical Research, University of Tasmania, Hobart 7005, Australia; charlotte.mckercher@utas.edu.au (C.M.); matthew.jose@utas.edu.au (M.J.); 4 Royal Hobart Hospital, University of Tasmania, GPO Box-1061, Hobart 7000, Australia; 5 School of Healthcare, University of Leeds, Leeds LS2 9JT, UK; S.T.R.Zaidi@leeds.ac.uk

**Keywords:** chronic kidney disease, medication appropriateness index, medication regimen complexity index, the elderly

## Abstract

This study aimed to examine the association between medication-related factors and risk of hospital readmission in older patients with chronic kidney disease (CKD). A retrospective analysis was conducted targeting older CKD (*n* = 204) patients admitted to an Australian hospital. Medication appropriateness (Medication Appropriateness Index; MAI), medication regimen complexity (number of medications and Medication Regimen Complexity Index; MRCI) and use of selected medication classes were exposure variables. Outcomes were occurrence of readmission within 30 and 90 days, and time to readmission within 90 days. Logistic and Cox hazards regression were used to identify factors associated with readmission. Overall, 50 patients (24%) were readmitted within 30 days, while 81 (40%) were readmitted within 90 days. Mean time to readmission within 90 days was 66 (SD 34) days. Medication appropriateness and regimen complexity were not independently associated with 30- or 90-day hospital readmissions in older adults with CKD, whereas use of renin-angiotensin blockers was associated with reduced occurrence of 30-day (adjusted OR 0.39; 95% CI 0.19–0.79) and 90-day readmissions (adjusted OR 0.45; 95% CI 0.24–0.84) and longer time to readmission within 90 days (adjusted HR 0.52; 95% CI 0.33–0.83). This finding highlights the importance of considering the potential benefits of individual medications during medication review in older CKD patients.

## 1. Introduction

Chronic kidney disease (CKD) is associated with a substantial risk of cardiovascular-related morbidity and mortality [1]. Conversely, cardiovascular diseases are also among the primary causes of morbidity and mortality in patients with CKD [2]. Therefore, pharmacological treatment in patients with CKD is largely directed at preventing and managing these cardiovascular problems. In addition to these comorbidities, CKD-related complications, such as anaemia and bone and mineral disorders, further complicate pharmacological approaches when treating these patients.

Multimorbidity in CKD is associated with higher medication burden and poorer survival [3]. Due to comorbidities and disease complications, the use of multiple medications in patients with CKD is often unavoidable, increasing the risk of exposure to medication-related problems that can lead to adverse drug events [4]. Medication-related problems are common causes of hospitalisation, mortality, and poorer quality of life in people with CKD [4,5]. Conversely, a number of medications, including sodium bicarbonate, erythropoiesis-stimulating agents, urate-lowering therapy, renin-angiotensin system (RAS) blockers, statins, and mineralocorticoid antagonists, are associated with improved outcomes in patients with CKD [6,7,8]. Therefore, the suboptimal and/or inappropriate use of such medications could potentially lead to poor patient and clinical outcomes.

Most studies examining medication use and outcomes in individuals with CKD have focussed on those with end-stage kidney disease (ESKD) [5,9,10,11], with a lack of studies targeting patients in earlier stages of the disease. Also, while previous studies have frequently reported the prevalence and type of inappropriate medications in CKD, evidence associating medication-related factors with risk of hospitalisation remains limited [12,13,14]. Finally, despite the reported clinical benefit of certain classes of medications in this patient group [6], there is inadequate information on the clinical impact of using preventive medications in older adults with CKD. Therefore, we aimed to investigate the association between medication-related factors (medication appropriateness, regimen complexity and the use of selected medications) and the occurrence of hospital readmission and time to readmission in hospitalised older patients with CKD.

## 2. Materials and Methods

### 2.1. Study Design, Participants and Data Collection

**Figure 1:**
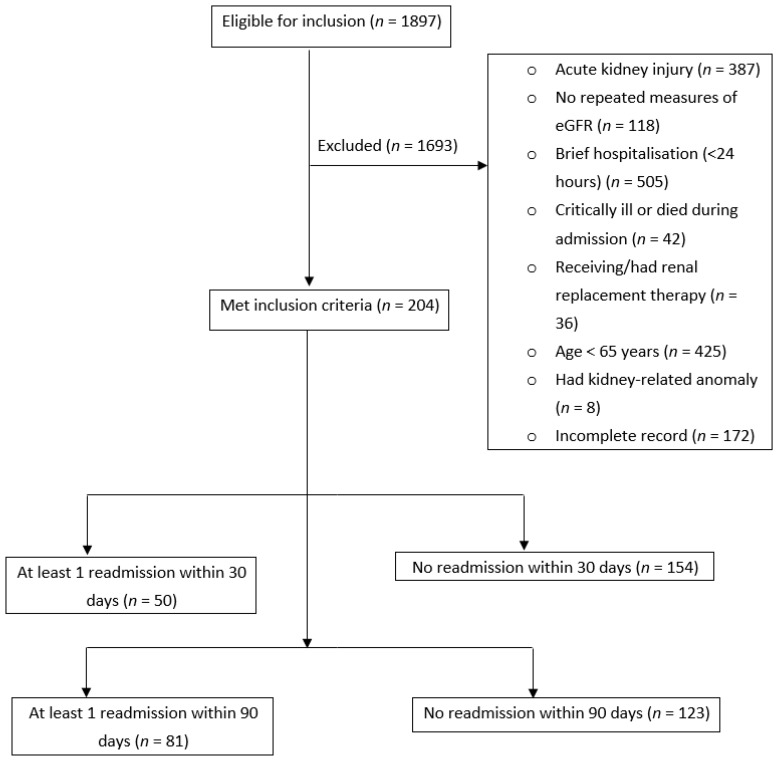
Flowchart of the inclusion process.

A retrospective study targeting 204 older adults (≥65 years) with CKD, consecutively admitted to a tertiary care hospital in Tasmania for any cause between 1 January and 30 June 2015 [15], was conducted. Out of the 1472 eligible older adults with an estimated glomerular filtration rate (eGFR) <60 mL/min/1.73 m^2^, we included 204 patients who fulfilled the inclusion criteria, i.e., had a documented diagnosis of CKD or repeated eGFR values between 15–60 mL/min/1.73 m^2^ reported for at least three months prior to admission and had not received any form of renal replacement therapy [16]. The eGFR values were estimated using the CKD Epidemiology Collaboration (CKD-EPI) [17] and are automatically reported along with requests for serum creatinine. Patients who stayed in hospital briefly (<24 h), with incomplete medical records and/or with acute kidney injury (AKI), alone or superimposed on CKD, were excluded. AKI was identified based on documentation (as noted in medical progress notes or discharge summaries) or a marked increase in serum creatinine (≥1.5 times the baseline value) [18]. People who were critically ill or died during the index hospitalisation were also excluded (Figure 1).

Demographic, laboratory, and clinical information for each patient was extracted using a state-wide digital medical record (DMR). Medications being used on a long-term basis, administered both regularly or as required, were recorded and coded using the Anatomical Therapeutic Classification (ATC) classification system of the World Health Organization [19]. Patient comorbidities and causes for the index admission and subsequent hospitalisations were coded using the *International Classification of Diseases*, 10th edition (ICD-10) [20].

### 2.2. Exposure Variables

Main exposure variables were medication-related factors, including medication regimen complexity (measured using the validated Medication Regimen Complexity Index (MRCI) [21] and the number of regularly taken medications), medication appropriateness (evaluated via the Medication Appropriateness Index; MAI) [22], and the use of selected medications at hospital discharge. The MRCI is a tool that measures an individual’s regimen complexity by taking into an account the dosage form, dosing frequency, and additional instructions. The MAI is an implicit measure of medication appropriateness containing 10 pharmacotherapeutic aspects: indication, effectiveness, dosage, directions and their feasibility, drug-drug and drug-disease interactions, duration, duplication and expense of medications. For practical reasons, we excluded two components of the MAI during the evaluation: the feasibility of directions and relative expense of medications [15,23]. Patient MAI scores were the summation of that of the individual medications, with higher scores reflecting a higher level of medication inappropriateness.

Given their benefit to CKD patients in general [24,25], the use of statins or RAS blockers at the index hospital discharge was included as an exposure variable in this study. Additionally, based on previous studies [6,11], the use of calcium channel blockers, beta blockers, diuretics, mineralocorticoid antagonists, and anticoagulants was also assessed during the analyses.

### 2.3. Outcome Variables

Outcomes of interest were (i) the occurrence of hospital readmission within 30 and 90 days of discharge and (ii) time to readmission in a 90-day follow-up period. These relatively short periods were chosen to minimise the possibility of significant changes in medication regimens of patients following their index hospitalisation.

### 2.4. Covariates

Renal function was measured using eGFR and serum creatinine values reported at the index hospital admission (at points closest to the date of admission). Comorbidity status was assessed at baseline using the original version of Charlson’s Comorbidity Index (CCI) [26], Given the well-established link between previous hospitalisations and hospital readmission [27], the number of hospitalisations in the six months preceding the index admission was also recorded as a covariate. Socioeconomic status of patients was calculated using the Index for Relative Socioeconomic Disadvantage [28], This index considers different variables to indicate the relative disadvantage of areas, with lower scores on this index reflecting a higher proportion of relatively disadvantaged people in an area.

### 2.5. Analyses

Descriptive statistics were reported using means (SD) or medians (IQR) depending on the normality of data distribution, which was assessed via visual inspection of histograms. Patient, laboratory, and clinical variables were compared in patients with or without readmission during the follow-up periods. For these comparisons, chi-square tests were used to examine the differences in categorical variables between these groups. Independent samples *t*-tests and Mann-Whitney *U* tests were applied to compare continuous variables, depending on the fulfilment of the assumption of normality of distribution.

To assess the association between medication-related variables at hospital discharge and 30- and 90-day readmissions, binary logistic regression was used, with effect sizes reported as odds ratios (ORs) and 95% confidence intervals (CIs). To determine the association between the medication-related factors and time to 90-day readmission, Cox proportional hazards regression was utilised, with effect sizes reported as hazard ratios (HRs) and 95% CIs. We employed two models in the Cox regression analysis: one partially adjusted for age, gender, and CCI (Model 1) and another one fully adjusted for the factors in Model 1 plus eGFR, the number of prior hospitalisations and discharge destinations (home versus residential care). Variables were included in the multivariate models based on either a *p* < 0.1 result on unadjusted analysis or a priori based on their relevance in predicting similar outcomes in previous studies [1,11,29]. For this analysis, the end of follow-up was set at 90 days after hospital discharge or date of death, whichever occurred first. Kaplan–Meier plots were also used for visual depiction of the difference in readmission risks within 90 days based on different MAI scores (categorised into quartiles as follows: 0–2; 3–5; 6–9 and 10–29) and the use of RAS blockers. Finally, to understand if people with multiple hospitalisations were medically more complex, we examined the relationship between the number of prior hospitalisations and medication-related variables at hospital admission, with results reported using Spearman’s coefficient. Analyses were performed using STATA, version 15.1 (StataCorp LLC, College Station, TX, USA). The study was conducted in accordance with the Declaration of Helsinki, and the Tasmanian Human Research Ethics Committee granted ethical approval for this study (H0016044).

## 3. Results

Overall, 204 older CKD patients (61% males) were included for analysis (Figure 1). Of these, 50 (24.5%) and 81 (40%) patients were readmitted at least once within 30 and 90 days of discharge from the index hospitalisation, respectively. The mean number of days to 90-day readmission was 66 (SD 34). Table 1 shows the baseline characteristics of participants. Additional laboratory and clinical information of the included patients are also presented in the attached Supplementary Material.

**Figure 2:**
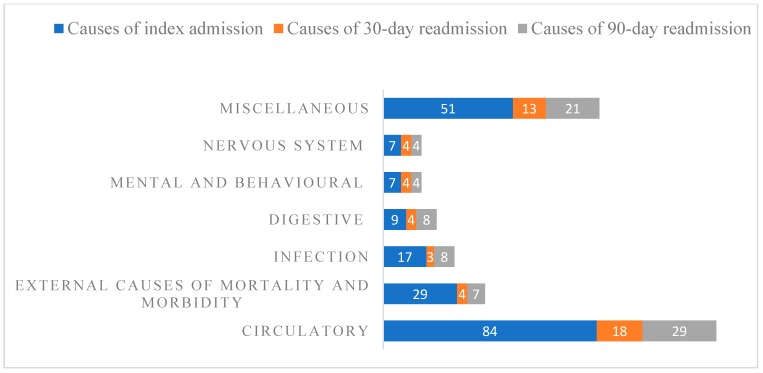
Causes for the index hospital admission and subsequent readmissions within 30 and 90 days, by ICD-10 classification (in frequencies).

The most common causes of index hospitalisation were diseases of the circulatory system (41%), external causes of morbidity and mortality (e.g., fall-related) (14%) and infections (8%). Diseases of the circulatory system also contributed to more than one-third of readmissions within 30 (36%) and 90 days (36%). (Figure 2) Half of the included patients were prescribed RAS blockers (51%), with an almost equivalent number taking statins (49.5%). Similarly, while half of the patients were on diuretics, nearly a third of them were prescribed calcium channel blockers (28%) and mineralocorticoid antagonists (25%).

Patients taking RAS blockers were relatively younger (mean [SD]: 80 [8] vs. 83 [7] years; *p* < 0.01) and had diabetes as a comorbidity (63% vs. 46%; *p* = 0.02) compared to those not on these medications. Additionally, most patients on RAS blockers had no hospitalisation record in the six months preceding the index admission (65%), with only 8.5% of them having three or more prior hospitalisations. However, users of RAS blockers were not significantly different in terms of gender (male: 52% vs. 48%), CCI (median [IQR]: 4 [2,3,4,5] vs. 4 [3,4,5]) and eGFR (mean [SD]: 36 [11] vs. 38 [9] mL/min/1.73 m^2^).

Table 1 shows that there was no significant difference in most of the medication-related variables among patients with or without hospital readmission within 30 days. MAI and MRCI were not significantly associated with the occurrence of readmission within 30 and 90 days on adjusted analyses (Table 2). However, the number of prior hospitalisations (in the six months before the index admission) significantly increased the risk of 30-day (OR 1.41 95% CI 1.05–1.90) and 90-day (OR 1.54 95% CI 1.15–2.10) readmissions after adjusting for age, gender, eGFR, CCI and the number of medications. The use of RAS blockers was associated with a reduced occurrence of readmission within both 30 (OR 0.39; 95% CI 0.19–0.79) and 90 days (OR 0.45 95% CI 0.24–0.84) after adjusting for the same variables.

**Figure 3:**
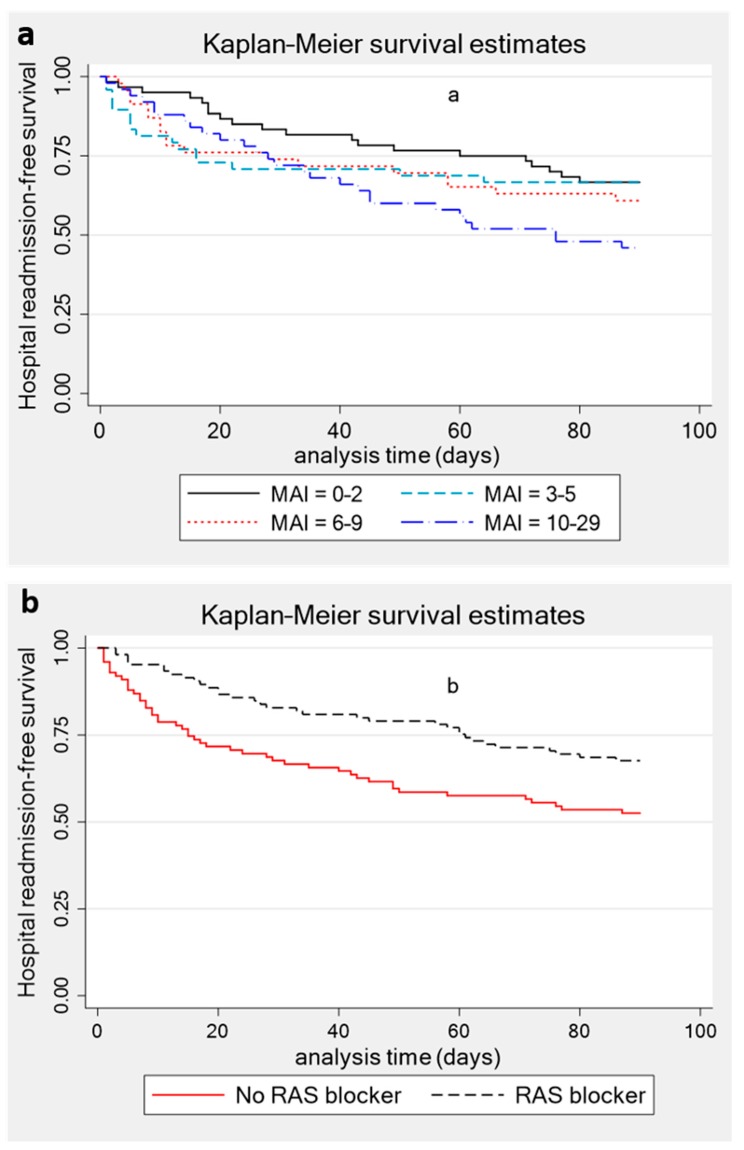
Kaplan-Meier hospital readmission-free survival by (**a**) quartiles of the MAI, and (**b**) the use of renin-angiotensin system (RAS) blocker.

Similarly, MAI and MRCI were not associated with time to 90-day readmission on fully adjusted models (Table 3). After adjusting for age, gender, CCI, eGFR and discharge destination, the number of prior hospitalisations was predictive of the timing of readmission within 90 days (HR 1.44; 95% CI 1.19–1.73). The Kaplan-Meier plots (Figure 3) illustrate that people in the highest quartile of the MAI had a relatively shorter time to readmission within 90 days compared to people in the lowest quartile (60 vs. 72 days; *p* < 0.05). The use of RAS blockers was associated with longer time to readmission within 90 days in both partially (HR 0.52 95% CI 0.33–0.83) and fully adjusted (HR 0.49; 95% CI 0.30–0.78) models (Table 3). The use of calcium channel blockers, beta blockers, diuretics, mineralocorticoid antagonists, and anticoagulants was not associated with hospital readmission in this patient group.

Finally, the number of prior hospitalisations was significantly associated with MRCI at the index hospital admission (Spearman’s *r* = 0.20; *p* = 0.02). However, it was not significantly associated with the number of medications (*r* = 0.14; *p* = 0.05) or the MAI (*r* = 0.10; *p* = 0.32) at the index admission.

## 4. Discussion

This study indicates that medication appropriateness and regimen complexity were not independent predictors of hospital readmission within 30 and 90 days in older adults with CKD. However, unadjusted results suggested that people with higher degree of medication inappropriateness and more complex regimens tended to be readmitted relatively sooner within 90 days of discharge. These findings suggest that medication inappropriateness and regimen complexity, even though not independently associated with readmission, were likely to reflect poorer health and multimorbidity, which may have contributed to the overall readmission rate in these patients.

Previous studies have reported conflicting results regarding the relationship between medication appropriateness and hospital readmission. For example, studies reported that a greater number of medications, but not inappropriate medication use, was associated with elevated risk of hospitalisation and death [12,30], with no differences observed in people with or without CKD [12]. Furthermore, a randomised trial aimed at improving medication therapy management in CKD patients (stage 3–5; not on dialysis) after hospital discharge did not translate to a reduced occurrence of readmission within 90 days [31]. In contrast, another study targeting general older adults showed that the use of inappropriate medications (measured using explicit measures) at hospital discharge was significantly associated with repeated hospital readmissions [32].

The number of hospitalisations in the six months prior to the index admission was strongly associated with hospital readmission within 30 and 90 days. Prior hospitalisation increased the odds of 30- and 90-day readmissions by 43% and 54%, respectively, and resulted in a significantly shorter time to readmission within 90 days. The association between previous hospitalisations and risks of subsequent readmission in older adults has previously been reported [14,27,33]. It would be anticipated that people with repeated hospitalisations are likely to be sicker and medically more complex than those with less frequent hospitalisations. This probably explains the significant correlation between the number of prior hospitalisations and medication regimen complexity at hospital admission in our study. Additionally, given that each hospital admission has the potential of causing a substantial change in the medication regimen [34], multiple hospitalisations are bound to result in frequent medication regimen changes in these patients. This, in turn, may affect medication management and adherence, especially in older adults with lower cognitive functioning or social support.

An important finding from this study was the association between the use of RAS blockers and lower risk of readmission, both within 30 and 90 days. This is interesting because these medications are the first-line treatment for hypertension in diabetic and nondiabetic CKD patients, especially those with proteinuria [25,35]. These medications are also an important part of pharmacotherapy in different cardiovascular conditions and their use is associated with lower cardiovascular events in people with CKD [36,37]. It is worth noting that the main reasons for index hospitalisation and hospital readmissions in our study were cardiovascular in nature. Therefore, in addition to their role in maintaining residual renal function [38], the cardioprotective effect of these medications appears to be of great importance in terms of reducing potential cardiovascular-related admissions. Consistent with our report, a longitudinal study that followed older adults with ESKD for up to three years reported an association between use of RAS blockers and lower risk of hospitalisation [11]. However, another study targeting a small number of older patients with stage 4 and 5 CKD (mean eGFR of 16.38 mL/min/1.73 m^2^) reported that the discontinuation of RAS blockers was associated with improved outcome [39]. Another large study targeting people with AKI also showed that the use of RAS blockers was linked to lower mortality but a higher risk of hospitalisation for renal causes, especially acute renal failure and hyperkalemia [40]. Of note, we found higher use of RAS blockers in the relatively younger and in those with no hospitalisation history, suggesting that these medications tended to be prescribed more commonly to relatively healthier individuals. This could be because frail individuals, such as those with frequent hospitalisations, may have a lower tolerance to the potential adverse effects of these medications, including worsening renal function, hyperkalaemia, and hypotension. While available data generally indicate the benefit of these medications in older CKD patients, the presence of proteinuria as well as the risk of progression and overall health should be considered when initiating RAS blockers [41].

Our study has some strengths and limitations. Access to data from the state-wide DMR has enabled us to accurately track patients’ hospitalisation records. Admission data in this study are therefore likely to be accurate and complete. The use of validated tools to evaluate medication appropriateness and regimen complexity is another strength of the study. We evaluated the association between medication-related factors at one point (discharge) and readmission after up to three months of follow-up. Our analysis, therefore, did not consider changes in medication regimens during the follow-up period. Finally, although we assessed the impact of medication-related factors, due to the retrospective nature of the study, we were not able to assess the level of medication adherence or ascertain the causality of the observed associations.

## 5. Conclusions

Medication appropriateness and regimen complexity were not independently associated with 30- and 90-day hospital readmissions in older adults with CKD. While there is a clear need for a larger prospective study, the significant association between the use of RAS blockers and reduced risk of 30- and 90-day readmissions suggests that these medications could be particularly beneficial in older adults with CKD.

## Figures and Tables

**Table 1:** Patient characteristics by 30- and 90-day readmissions.

Characteristics	Total (*n* = 204)	30-Day Readmission			90-Day Readmission		
		Yes (*n* = 50)	No (*n* = 154)	*p*	Yes (*n* = 81)	No (*n* = 123)	*p*
Age (years), mean (SD)	82 (7.6)	81 (7.3)	82 (7.7)	0.61	81 (7.4)	82 (7.8)	0.37
Male gender, *n* (%)	125 (61)	36 (72)	89 (58)	0.07	54 (67)	71 (58)	0.20
SBP (>140 mm Hg), *n* (%)	84 (41)	20 (40)	64 (42)	0.85	36 (44)	48 (39)	0.44
Serum creatinine (μmol/L), median (IQR)	134 (113–162)	142 (119–183)	134 (110–162)	0.11	128 (115–164)	138 (113–162)	0.16
eGFR (mL/min/1.73 m^2^), mean (SD)	37 (10)	36 (12)	37 (9.6)	0.16	37 (9)	37 (11)	0.77
CCI, median (IQR)	4 (3–5)	4 (3–5)	4 (2–5)	0.35	4 (2–5)	4 (2–5)	0.17
CCI (>3), *n* (%)	157 (77)	41 (82)	116 (75)	0.33	68 (84)	89 (72)	0.05
No. of medications at admission, median (IQR)	10 (7–13)	10 (6–13)	10 (7–12)	0.32	10 (7–12)	9 (6–13)	0.18
No. of medications at discharge, median (IQR)	10 (7–13)	10 (7–13)	10 (7–13)	0.24	11 (7–13)	9 (6–12)	0.02
MRCI at admission, median (IQR)	25 (17–33)	28 (21–34)	24 (16–33)	0.09	27 (20–34)	23 (15–32)	0.04
MRCI at discharge, median (IQR)	27 (20–35)	30 (21–36)	26 (18–34)	0.12	30 (22–37)	26 (17–34)	0.01
MAI at admission, median (IQR)	6 (3–12)	6 (4–13)	6 (3–11)	0.49	7 (4–12)	6 (3–11)	0.08
MAI at discharge, median (IQR)	5 (2–9)	6 (3–10.5)	4.5 (2–9)	0.23	7 (2.5–12)	4 (2–8)	0.03
Use of different medications, *n* (%)							
RAS blockers	105 (51)	18 (36)	87 (56)	0.01	35 (43)	70 (57)	0.05
Statins	101 (49.5)	26 (52)	75 (49)	0.68	38 (47)	63 (51)	0.55
Calcium channel blockers	58 (28)	14 (28)	44 (29)	0.93	23 (28)	35 (28)	0.99
Beta blockers	94 (46)	23 (46)	71 (46)	0.99	39 (48)	55 (45)	0.63
Diuretics	104 (51)	26 (52)	78 (51)	0.87	44 (54)	60 (48)	0.44
Anticoagulants	53 (26)	11 (22)	42 (27)	0.46	18 (22)	35 (28)	0.32
Aldosterone antagonist	32 (16)	10 (20)	22 (14)	0.33	15 (18)	17 (14)	0.37
Primary cause of hospitalisation, *n* (%)				0.71			0.63
Cardiovascular	80 (39.2)	22 (44)	58 (38)		35 (43)	45 (37)	
Infection	25 (12.2)	6 (12)	19 (12)		9 (11)	16 (13)	
Other	99 (48.5)	22 (44)	77 (50)		37 (46)	62 (50)	
Prior admission(s) in six months before, *n* (%)	101 (49.5)	27 (54)	74 (48)	0.46	49 (60)	52 (42)	0.01
Discharge destination, *n* (%)				0.18			0.09
Home	162 (79.4)	43 (86)	119 (77)		69 (43)	93 (57)	
Residential care	42 (20.1)	7 (14)	35 (23)		12 (29)	30 (71)	
IRSD (lowest quartile)	51 (12)	14 (28)	37 (24)	0.59	22 (27)	29 (23.5)	0.68

Abbreviations: ALP, alkaline phosphatase; ALT, alanine aminotransferase; AST, aspartate aminotransferase; CCI, Charlson’s comorbidity index; eGFR, estimated glomerular filtration rate; IQR, interquartile range; IRSD, index of relative socioeconomic disadvantage; IU, international unit; MAI, Medication Appropriateness Index; MRCI, medication regimen complexity index; RAS, renin angiotensin system; SBP, systolic blood pressure; SD, standard deviation.

**Table 2:** Logistic regression for medication-related factors and occurrence of readmission within 30 and 90 days.

**a Readmission within 30 Days**		
	Unadjusted ORs (95% CIs)	Adjusted ORs (95% CIs) ^¶^
MAI	1.03 (0.97–1.09)	1.01 (0.94–1.07)
MRCI	1.21 (0.94–1.55)	1.24 (0.95–1.63)
Use of RAS blockers	0.43 (0.22–0.84)	(0.19–0.79)
**b Readmission within 90 Days**		
MAI	1.07 (1.01–1.12)	1.06 (1.00–1.12)
MRCI	1.33 (1.06–1.68)	1.31 (0.99–1.72)
Use of RAS blockers	0.58 (0.33–1.01)	0.45 (0.24–0.84)

Abbreviations: MAI, Medication Appropriateness Index; MRCI, medication regimen complexity index; ORs, odds ratios; RAS, renin-angiotensin system ^¶^ Analysis adjusted for age, gender, eGFR, Charlson’s Comorbidity Index, prior admissions and the number of medications.

**Table 3:** Cox proportional hazards regression for medication-related factors and time to readmission within 90 days.

	Unadjusted HRs (95% CIs)	Adjusted HRs (95% CIs)
Model 1		
MAI	1.04 (1.01–1.08)	1.04 (1.01–1.08)
MRCI	1.18 (1.01–1.39)	1.23 (1.03–1.47)
Use of RAS blockers	0.57 (0.38–0.89)	0.52 (0.33–0.83)
Model 2		
MAI	1.04 (1.01–1.08)	1.03 (0.99–1.08)
MRCI	1.18 (1.01–1.39)	1.16 (0.96–1.39)
Use of RAS blockers	0.57 (0.38–0.89)	0.49 (0.30–0.78)

Abbreviations: HRs, hazard ratios; MAI, Medication Appropriateness Index; MRCI, medication regimen complexity index; RAS, renin-angiotensin system. Model 1: Analyses adjusted for age, gender and Charlson’s Comorbidity Index. Model 2: Analyses adjusted for factors in Model 1 plus eGFR, prior admissions and discharge destination.

## Supplementary Materials

The following are available online at https://www.mdpi.com/2077-0383/8/3/395/s1, Table S1: Additional laboratory and clinical characteristics of the included patients by readmission status (*n* = 204).
