# Supplementary material for: Medication-Related Factors and Hospital Readmission in Older Adults with Chronic Kidney Disease
Source: J Clin Med. 2019 Mar 21;8(3):395. doi: 10.3390/jcm8030395 (PMC6462973; doi:10.3390/jcm8030395)
Supplement: Supplementary file 1 [file jcm-08-00395-s001.pdf]

Supplementary 1: Additional laboratory and clinical characteristics of the included patients by readmission status (*n* = 204)

		30–day readmission			90–day readmission		
Characteristics	Total (*n* = 204)	Yes (*n* = 50)	No (*n* = 154)	P	Yes (*n* = 81)	No (*n* = 123)	P
Hemoglobin (g/L), median (IQR)	118 (106–134)	117 (103–133)	118 (108–134)	0.54	118 (105–131)	119 (106–135)	0.68
Serum albumin (g/L), median (IQR)	32 (29–36)	32 (29–37)	32 (29–36)	0.66	33 (29–37)	32 (29–37)	0.34
Elevated Ca (>2.55 mmol/L), n (%)	24 (11.2)	5 (10)	44 (28)	0.01	6 (7.4)	18 (14.6)	0.12
Elevated PO_4 (>1.50 mmol/L), n (%)	15 (18.5)	4 (8)	11 (7)	0.84	8 (10)	7 (5.7)	0.26
Sodium (mmol/L), median (IQR)	142 (137–144)	140 (136–142)	139 (137–142)	0.42	142 (140–142)	139 (137–142)	0.92
Potassium (mmol/L), median (IQR)	5.2 (4.6–5.6)	4.7 (4.2–5.2)	4.6 (4.2–5.2)	0.95	4.6 (4.2–5.1)	4.6 (4.2–5.2)	0.59
ALT (IU/L), median (IQR)	16.5 (11–26)	19.5 (12–30)	16 (11–26)	0.11	16 (12–26)	17 (11–27)	0.56
ALP (IU/L), median (IQR)	88.5 (72–114)	92.5 (67–125)	88 (73–111)	0.44	89 (68–117)	88 (74–113)	0.82
AST (IU/L), median (IQR)	23 (16–52)	23 (16–35)	23 (15–32)	0.47	22 (16–30)	23 (16–33)	0.60
Common comorbidities, n (%)							
Hypertension,	145 (71)	30 (60)	115 (75)	0.05	55 (68)	90 (73)	0.42
Diabetes	70 (34)	22 (44)	48 (31)	0.10	30 (37)	40 (32.5)	0.51
Atrial fibrillation	66 (32)	16 (32)	50 (32)	0.95	25 (31)	41 (33)	0.71
Heart failure	49 (24)	11 (22)	38 (25)	0.70	20 (25)	29 (24)	0.85
Length of hospitalisation, median (IQR)	4 (2–8)	4 (2–8)	4 (3–8)	0.85	4 (2–8)	4 (3–8)	0.89
Primary cause of hospitalisation, n (%)				0.71			0.63
Cardiovascular	80 (39.2)	22 (44)	58 (38)		35 (43)	45 (37)	
Infection	25 (12.2)	6 (12)	19 (12)		9 (11)	16 (13)	
Other	99 (48.5)	22 (44)	77 (50)		37 (46)	62 (50)	

Abbreviations: ALP, alkaline phosphatase; ALT, alanine aminotransferase; AST, aspartate aminotransferase;
